# Secondary Acrocyanosis in a Paraplegic Patient With Spinal Cord Injury

**DOI:** 10.7759/cureus.29420

**Published:** 2022-09-21

**Authors:** Juan F Ruiz-Rodríguez, Ricardo J Fernández-de Thomas, Orlando De Jesus

**Affiliations:** 1 Neurosurgery, University of Puerto Rico, Medical Sciences Campus, San Juan, PRI

**Keywords:** vasospastic disorders, sympathetic denervation, spinal cord injury, paraplegia, acrosyndromes, acrocyanosis

## Abstract

Secondary acrocyanosis after spinal cord injury is extremely rare. We describe a case with secondary acrocyanosis in a complete T12 paraplegic patient. A 41-year-old man with complete T12 paraplegia after a gunshot wound to the thoracic spine 20 years prior presented with a four-month history of bilateral foot bluish discoloration precipitated when he sat with his legs down, improving rapidly after a few minutes of leg elevation. Changes in the skin color of the lower extremities were evaluated in the seated position for two hours. The skin color became darker, progressing to a bluish discoloration through the entire length of the legs. After two hours, the feet and most of the legs appeared deep purple. The color of the legs returned to their baseline three minutes later after the patient was placed supine in the bed. The diagnosis of secondary acrocyanosis due to the T12 spinal cord injury was established based on the physical examination and ancillary tests showing no peripheral ischemia. Other causes of secondary acrocyanosis were excluded during the work-up. This report presents the first case of a paraplegic patient with spinal cord injury presenting secondary acrocyanosis.

## Introduction

Acrocyanosis is a painless discoloration of different shades of blue in the distal parts of the body marked by symmetry, relative persistence of the skin color changes, and frequent association with hyperhidrosis of hands and feet [1-3]. Acrocyanosis is one of the three main vascular acrosyndromes; the other two are Raynaud’s syndrome and erythromelalgia. There are no universally accepted diagnostic criteria for acrocyanosis, erythromelalgia, and Raynaud’s syndrome. Thus, the clinical diagnosis relies primarily on history and physical examination [4]. Acrocyanosis is exacerbated by the extremity being in a dependent position and usually resolves when placed horizontally [4]. A distinction should be made between primary acrocyanosis without detectable underlying disease and secondary acrocyanosis with a specific underlying condition. Secondary acrocyanosis due to spinal cord injury is extremely rare, with only two cases reported in the English literature attributed to cervical spine injuries [5,6].

## Case presentation

A 41-year-old man with complete T12 paraplegia after a gunshot wound to the spine 20 years prior presented with a four-month history of bilateral foot discoloration and swelling. The discoloration and swelling precipitated when he sat with his legs down and improved significantly with a few minutes of leg elevation. The symptoms were not triggered by cold weather, cold exposure, anxiety, or emotional distress. He did not experience similar symptoms in the upper extremities. The patient denied fever, fatigue, recent systemic infections, cough, hemoptysis, chest pain, weight loss, easy bruising, systemic rashes, or recent trauma to extremities. The patient had no history of autoimmune diseases, peripheral vascular disease, coagulation disorders, deep vein thrombosis, or other chronic diseases. His family medical history was unremarkable. The patient has been an inmate at a correctional facility since he received the gunshot wound to the spine. He denied smoking, alcohol use, or illicit drug use.

**Figure 1:**
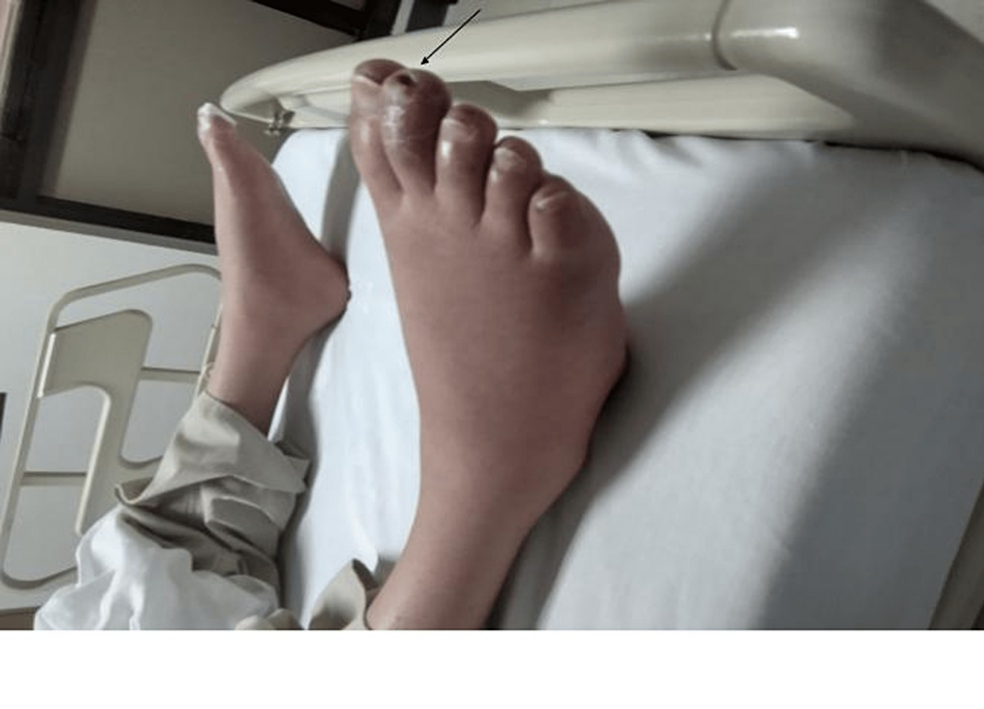
The patient’s lower extremities supine on the bed show no skin discoloration. Thickened scaly skin of distal feet with scabbing at the tips of some toes is noted (black arrow).

The vital signs were within normal range, and oxygen saturation was 98% at room air. His neurologic examination showed a complete motor deficit of the lower extremities with a complete sensory deficit just below the umbilicus. He was areflexic on the lower extremities. There was no rectal tone. The severity of the injury was compatible with a T12 complete motor and sensory deficit. There was marked atrophy of the lower extremity musculature. He had normal femoral, popliteal, and pedal pulses bilaterally. On examination of the lower extremities in the supine position, he had thickened scaly skin of distal feet with a few punctate erythematous lesions on the plantar aspect of the toes with scabbing at the tips of some toes without skin discoloration of the legs or feet (Figure 1).

**Figure 2:**
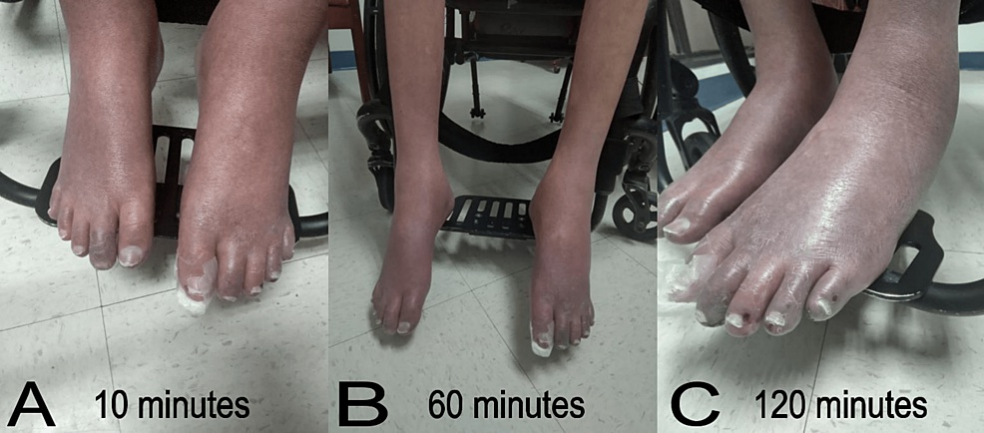
Changes in the skin color of the patient's lower extremities were evaluated in a seated position for two hours at (A) 10 minutes, (B) 60 minutes, and (C) 120 minutes.

Evaluation with the legs hanging off the bed showed visible blue/purple discoloration on the distal lower extremities with swollen feet. Changes in skin color were evaluated in a seated position for two hours. The changes in the skin color were symmetrical, and the arterial pulses remained normal during the examination. At 15 minutes, the skin color became darker, and the discoloration started just above the ankles and extended to both feet. At 60 minutes, the bluish discoloration darkened and progressed through the entire length of the leg, associated with mild pitting edema. At two hours, the feet and most of the legs appeared deep purple (Figure 2).

**Figure 3:**
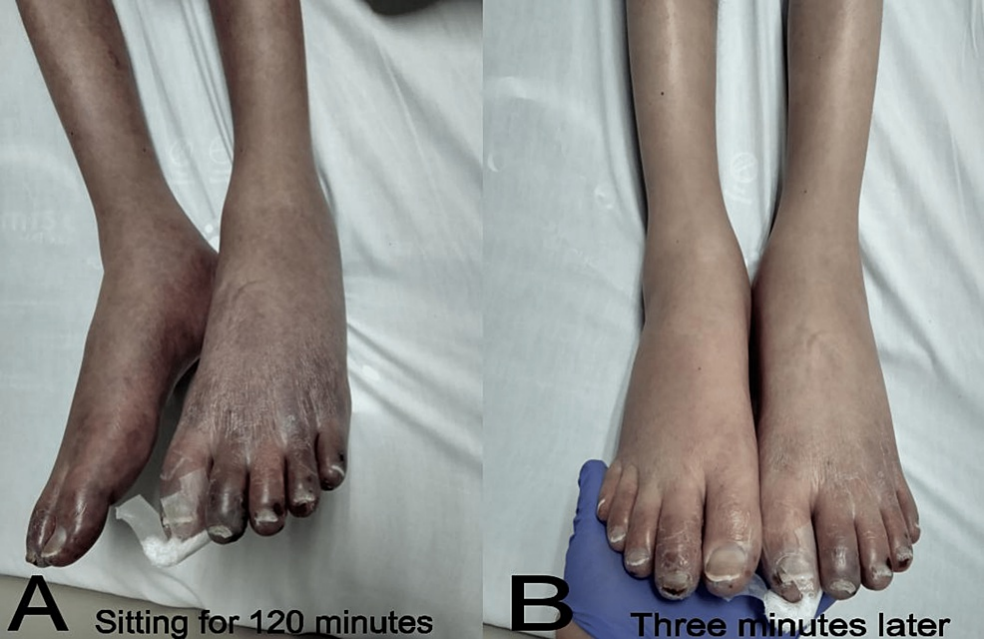
(A) Patient’s lower extremities supine at the bed immediately after sitting for 120 minutes, showing deep purple discoloration. (B) After the legs were extended for three minutes, there was a significant improvement in the discoloration.

After two hours, the patient was placed back on the bed, and three minutes later, the color of the legs returned to their baseline (Figure 3).

A computer tomographic scan and angiography of lower extremities showed no evidence of arterial narrowing, obstruction, or identifiable masses. The computer tomographic scan and angiography of the chest and abdomen revealed scattered atherosclerotic plaques on the abdominal aorta and mild narrowing of the celiac trunk without significant stenosis. The comprehensive metabolic panel, complete blood count, and platelet count were within normal limits. The D-dimer test was normal. The toxicology screen was negative. The patient had negative antinuclear antibody test, anti-ds DNA, anti-Scl-70, anti-beta-2 glycoprotein 1, anti-smooth muscle, anti-mitochondrial, anti-cardiolipin, antineutrophil cytoplasmic antibody test, and serum cryoglobulins. Inflammatory markers C3, C4, and rheumatologic factor were negative. Blood cultures were negative. Hepatitis B antibody, hepatitis C antibody, S-antigen, Mycoplasma pneumoniae Immunoglobulin M (IgM), HIV test, and COVID-19 antigen were all negative. Tests were negative for protein S, protein C, antithrombin III, factor V Leiden, and lupus anticoagulant. A portable ultrasound bedside evaluation revealed adequate arterial blood flow on both lower extremities (right ankle-brachial index = 0.95 and left ankle-brachial index = 1.47). There was no evidence of deep vein thrombosis or venous obstruction. A bedside echocardiogram showed no evidence of vegetations and no atrial or ventricular thrombi.

A calcium channel blocker trial was given at the beginning of his hospitalization, hoping the patient would respond if it was Reynaud’s syndrome. However, there was no improvement. As the symptoms significantly improved with position changes of the lower extremity, the patient was encouraged to use the wheelchair’s leg supports to elevate the legs near 180 degrees when sitting in his wheelchair. He was discharged with a follow-up duplex ultrasound and evaluation at the clinic. Three months later, the duplex ultrasound showed adequate arterial blood flow on both lower extremities. On the examination, dorsalis pedis and posterior tibial artery pulses were normal bilaterally. The lower extremity discoloration persisted when the patient sat with the legs down.

## Discussion

The principal differential in this paraplegic patient was secondary acrocyanosis caused by the T12 spinal cord injury. The lack of autonomic vascular venous tone and chronic loss of leg muscle mass secondary to the patient’s spinal cord injury caused venous stasis at the lower extremities in the dependent position. The impaired sympathetic input to the lower extremities contributed to increased capacitance and decreased return of small and large venous vessels in the legs that became pronounced with a long seated position. A complete work-up was performed to exclude an atypical presentation of a more common condition associated with extremity discoloration and digital ischemia. Reynaud’s syndrome can present with digital discoloration and may lead to ischemic tissue loss. There were essential differences between our patient’s symptoms and those commonly associated with Reynaud’s syndrome. The patient’s symptoms were not episodic, not provoked by cold exposure or stress, did not involve the upper extremities, and the discoloration was not limited to the distal parts of the extremity. Reynaud’s syndrome would not explain the direct association between the patient’s symptoms and improvement with modifications in the position of the extremities. Our patient’s blue/purple discoloration of lower extremities was reversed within three minutes of a change in position. Erythromelalgia is characterized by intense pain in and warmth, and redness of the affected extremity. Although our patient is incapable of feeling pain in the lower extremities due to the complete spinal cord injury, warmth and redness of the affected extremity were absent.

Additional causes of secondary acrocyanosis are extensive; however, many were ruled out by the patient’s history, physical examination, laboratory reports, and diagnostic imaging tests. Vasculitis, collagen diseases, cryoglobulinemia, and other immunologic or rheumatologic conditions were less likely based on his laboratory test results and the lack of additional signs/symptoms. Acute or chronic ischemia was not expected to cause the patient's current presentation, as the ultrasound demonstrated adequate arterial blood flow on both lower extremities. There was no evidence of deep vein thrombosis or obstructed arterial vessels. Laboratory tests did not identify hypercoagulable syndromes such as antiphospholipid syndrome or factor V Leiden thrombophilia. Subacute/chronic endocarditis or other infectious etiologies were also possible etiologies; however, the patient was afebrile, had no murmur, negative blood cultures, and no vegetations on the echocardiogram. Pulmonary causes were excluded based on the computed tomographic scan results. Buerger’s disease was excluded as there was no history of smoking. Postural tachycardia syndrome and orthostatic intolerance were unlikely as the patient did not present dizziness or tachycardia in the sitting position. Secondary acrocyanosis associated with medications was excluded after reviewing the patient’s medication history.

Acrocyanosis demonstrates significant skin color changes with the extremity in a dependent position, resolving when elevated [1-4]. Digit color normalizes when the involved extremity is transferred from the dependent to the horizontal position. Acrocyanosis disappears with the elevation of the hands above the head, arguing against venous obstruction and supporting venous dilatation. Pain or significant discomfort is uncommon in acrocyanosis [1]. Secondary acrocyanosis can affect digits asymmetrically and sometimes is associated with pain and tissue damage [1]. It is postulated that acrocyanosis involves excessive venous pooling secondary to anatomical or functional sympathetic denervation, similar to orthostatic hypotension [6-8]. The major vasculature beds in the gut and lower extremities are controlled by the more caudal T5-L2 spinal sympathetic neurons [9]. A spinal cord injury frequently produces sympathetic denervation. In patients with long-standing spinal cord injury, venous capacity and compliance are reduced in the lower extremities [6,10,11]. The loss of sympathetic vasomotor tone is responsible for the reduction in venous vascular function as the walls of the veins contain smooth muscle innervated by the sympathetic nervous system [7,11]. The venous emptying rate is significantly reduced in patients with spinal cord injuries [11]. In the patient with spinal cord injury, secondary acrocyanosis of the lower extremities may result from venous dilation and pooling due to the loss of normal sympathetic vasomotor tone in the abdomen and extremities, commonly seen after a mid-thoracic or cervical cord lesion [6].

Secondary acrocyanosis due to cervical spinal cord injury has been described in only two patients. The first reported case described a patient with an incomplete C5-C6 spinal cord injury sustained 21 years before the presentation of secondary acrocyanosis [5]. The second reported case presented a C5 quadriplegic patient presenting secondary acrocyanosis 19 years after a diving accident [6]. Our case is the first to report secondary acrocyanosis in a paraplegic patient with spinal cord injury, as all prior reported cases involved the cervical spine. There is no effective therapy for primary acrocyanosis, but secondary forms can sometimes be treated by addressing the underlying condition. In the report of the patient with incomplete C5-C6 spinal cord injury, neuromuscular electrostimulation of the legs improved the cutaneous symptoms produced by secondary acrocyanosis [5].

## Conclusions

This case report demonstrated that secondary acrocyanosis could be associated with paraplegia. A thoracic spinal cord injury may cause secondary acrocyanosis due to the loss of normal sympathetic vasomotor tone to the lower extremities. The symptoms of acrocyanosis are exacerbated with the extremity in a dependent position, usually resolving when placed in a horizontal position.
